# The Potential Significance of ABO Genotyping for Donor Selection in Kidney Transplantation

**DOI:** 10.3389/fimmu.2020.608716

**Published:** 2020-11-19

**Authors:** Yi Zhou, Yuchen Wang, Haiqiang Ni, Wenfeng Deng, Ding Liu, Jian Xu, Naiqian Cui, Yihan Wu, Shaojie Fu, Lulu Xiao, Hailiang Liu, Ka Qi, Shaoqing Wang, Fu Xiong, Yun Miao

**Affiliations:** ^1^ Division of Transplantation, Nanfang Hospital, Southern Medical University, Guangzhou, China; ^2^ Division of Transplantation, Zhujiang Hospital, Southern Medical University, Guangzhou, China; ^3^ Department of Medical Genetics, School of Basic Medical Sciences, Southern Medical University, Guangzhou, China; ^4^ Hemodialysis Center, Qinhuangdao Charity Hospital, Qinhuangdao, China; ^5^ Nephrology Department, The First Affiliated Hospital of Chengdu Medical College, Chengdu, China

**Keywords:** ABO subtype, genotype, donor selection, serologic testing, kidney transplantation

## Abstract

**Background:**

The ABO blood group system is clinically important in kidney transplantation, but ABO genotyping fails to attract sufficient attention in some countries and regions. We identified one case of early graft dysfunction due to an ABO genotype mismatch. Here, we performed ABO genotyping in blood samples, analyzed grouping discrepancies, and investigated the weak A subgroup frequency in kidney transplantation candidates.

**Methods:**

Blood samples from 302 uremic patients with grouping discrepancies and 356 uremic patients with type A blood were analyzed using standard serologic serotyping techniques. The ABO genotypes and alleles were analyzed by polymerase chain reaction sequence-specific primer (PCR-SSP) and sequence-based typing (PCR-SBT).

**Results:**

All 302 uremic patients with grouping discrepancies carried weak ABO subgroup alleles and 77.48% carried irregular ABO antibodies. The discrepancy rate between serotyping and genotyping was 42.38%, and the mismatching rate of donor selection according to serotype reached 88.74%. And 2.53% of 356 uremic patients with type A blood were determined to be in the weak A subgroup, which was a higher percentage than that observed in the healthy Chinese population (0.53%) by serological screening, but much lower than that observed in Caucasians (20%).

**Conclusion:**

We revealed the high risk of blood type misjudgment and genetically ABO-mismatched transplantation if serological test was performed only in blood-group typing. Improved precision of ABO genotyping is crucial for successful kidney transplantation and reasonable organ allocation.

## Introduction

The ABO blood group is the most clinically relevant blood group system in transplant medicine and it is genetically complicated with more than 200 alleles (1, 2). The ABO gene is located on the 34.1–34.2 area of human chromosome 9; the gene contains seven exons and six introns, and the coding region consists of 1065 bases (3). Variants in the ABO gene can lead to different blood group-specific glycosyltransferases, thus giving rise to subgroups within the blood system (4). Weak ABO subgroups refer to phenotypes with weak expression of A or B antigen compared to the common ABO alleles (ABO*A1.01, ABO*A1.02, ABO*B1.01, ABO*O01.01, and ABO*O01.02) (5).

ABO antigens are widely expressed on the membranes of red blood cells and tissue cells, as well as in saliva and body fluids (6, 7). There is a higher risk of graft loss in ABO-incompatible (ABOi) kidney transplantation (8, 9) since stimulated antibodies can bind directly to blood group antigens on the renal endothelial surface and cause acute rejection (AR). This may also cause subsequent platelet aggregation by activating complement response in conjunction with vascular endothelial cells, leading to chronic rejection (CR) and, ultimately, allograft failure (10).

An update to the Organ Procurement and Transplantation Network policy stipulates dual confirmation of the donor A or AB subtype. This policy permits A2-to-O and A2B-to-B transplantation in order to shorten the waiting time for group O and group B recipients, respectively (11–13). However, in many countries and regions, serological typing is the only criterion for ABO blood grouping for kidney transplantation (14, 15). We revealed one clinical case of unexplained and irreversible early graft dysfunction in a serologically matched pair, and it was verified by genotyping as A1-to-A2 transplantation.

In this study, we performed ABO gene detection of blood samples with grouping discrepancies retrospectively. We investigated the frequency of the weak A subgroup in type A candidates to explore the significance of ABO genotyping for precise donor selection in kidney transplantation.

## Materials and Methods

### Samples

We retrospectively tested blood samples of a total of 302 kidney transplantation candidates who had never received a transplant reserved from Nanfang Hospital (Guangzhou, China), Qinhuangdao Charity Hospital (Qinhuangdao, China), and the First Affiliated Hospital of Chengdu Medical College (Chengdu, China) between January 2015 and December 2017. We also investigated 356 blood samples in the serological A group for subtype classification between January 2018 and October 2019 in Nanfang Hospital. Informed consent was obtained from patients, and the study was approved by the Ethics Committee of Nanfang Hospital (NFEC-2019-172), Southern Medical University.

### ABO Blood Group Testing

ABO serology of the samples was determined by monoclonal anti-A, anti-B, anti-AB, and anti-H; polyclonal anti-A, anti-B, and anti-AB reagents (Brother Biotech, Changchun, China) according to the manufacturer’s protocol; and an ABO red blood cell (RBC) kit for reverse typing. The intensity of microcolumn agglutination reaction (±, +, ++, +++, ++++) was determined by trained staff according to the comparison card provided by the reagent manufacturer.

### DNA Preparation and Polymerase Chain Reaction Amplification

Genomic DNA was extracted from 0.5 ml EDTA-anticoagulated peripheral blood using a genomic DNA purification kit (Tianjin Super Biotechnology Developing Co., Ltd., Tianjin, China). The primers for polymerase chain reaction (PCR) were designed according to the published ABO gene sequence (GenBank Accession Number NC_000009.12), as listed in **Supplementary Table 1**. The PCR reaction conditions were as follows: 96°C for 2 min (denaturation); 96°C for 20 s followed by 68°C for 60 s for 5 cycles; 96°C for 20 s, 65°C for 50 s, and 72°C for 45 s for 10 cycles; 96°C for 20 s, 62°C for 50 s, and 72°C for 45 s for 22 cycles; 72°C for 2 min (extension); and 4°C for thermal insulation. The PCR products were separated by 2.5% agarose gel electrophoresis at 150 V for 15 to 20 min. Electrophoresis was continued until the control band separated completely from the positive band, and results were observed using an ultraviolet imaging system (GenoSens1860, Clinx, Shanghai, China).

### PCR Sequence-Specific Primer

The polymerase chain reaction sequence-specific primer (PCR-SSP) subtype blood genotyping kit (Tianjin Super Biotechnology Developing Co., Ltd., Tianjin) was used according to the manufacture’s protocol. The mixture contained 110 μl dNTP-Buffer, 0.9 μl TaqDNA polymerase (5 units/μl, Promega, USA), and 10 μl sample DNA. The volume of amplification reaction system in each well was 10 μl (11 wells per person).

### DNA Sequencing for Full the Coding Region of the ABO Gene

The PCR amplification products were purified and then sequenced using Sanger’s dideoxy termination method with a unidirectional specific primer which were the same as those of PCR (**Supplementary Table 1**). We analyzed the sequence data with BIO-Mutation™, and all obtained nucleotides sequences were compared with standard ABO polymorphism site sequences from the database red blood cells (dbRBC) of NCBI.

### Nomenclature of Mutations and ABO Alleles

The ABO variants and alleles were named according to the nomenclature used by the International Society of Blood Transfusion (ISBT). If an ISBT allele name was not available, a name in the original literature was used in square brackets.

## Results

### Clinical Characteristics of 302 Kidney Transplant Candidates

Among 302 kidney transplant candidates, 192 (63.58%) were male patients. A total of 101 (33.44%) candidates had previously been pregnant; and 93 (30.79%) had a prior blood transfusion. Twelve candidates had comorbid condition including hematologic diseases (n=8, 2.65%) and tumors (n=4, 1.32%).

### Genotype Distribution in 302 Transplant Candidates

**Figure 1:**
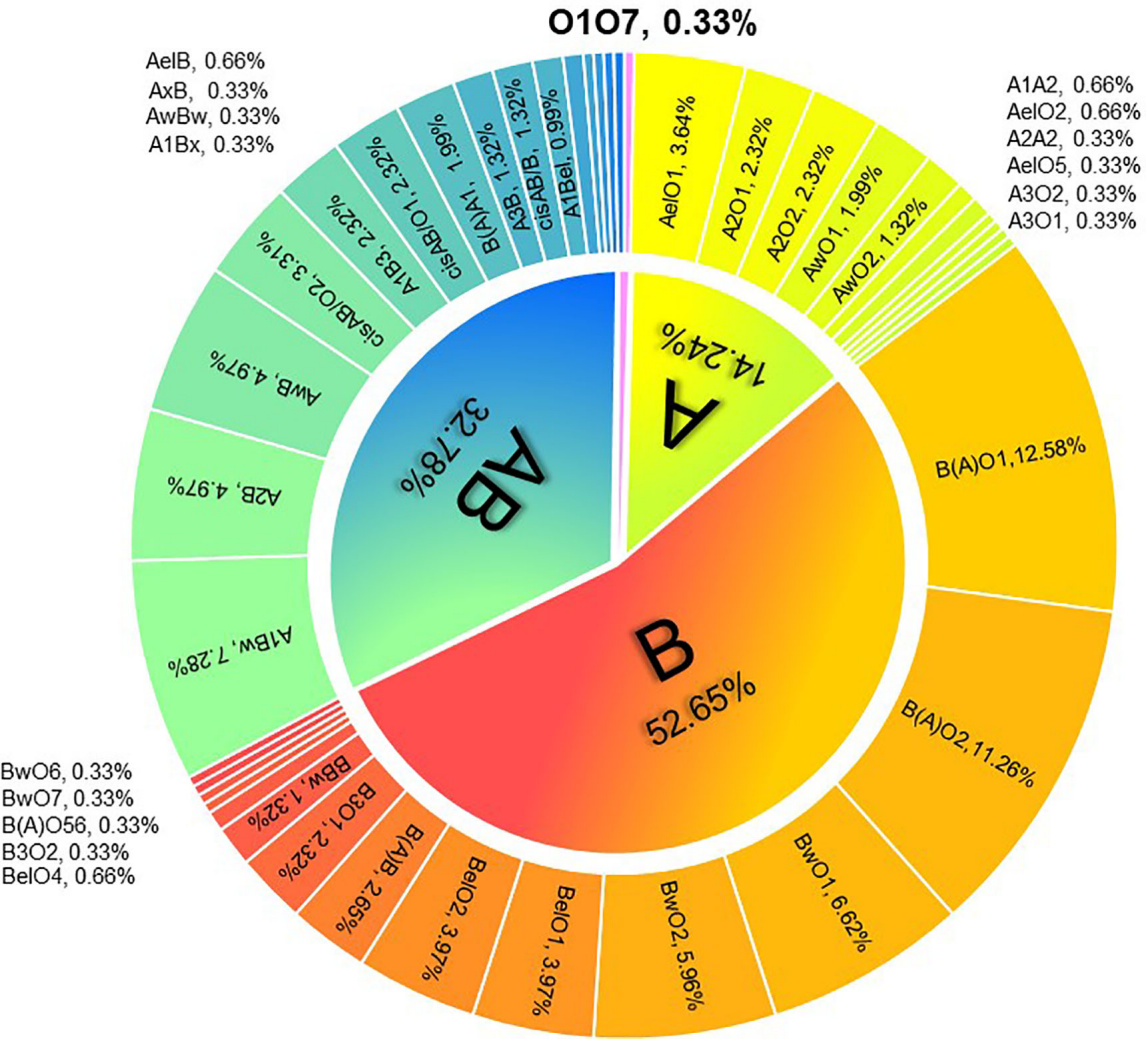
Distribution of ABO genotypes in 302 kidney transplantation candidates with ABO grouping discrepancies.

ABO serotyping with monoclonal reagents identified groups O (n=19), A (n=43), B (n=63), and AB (n=177) while the genotypes were groups O (n=1), A (n=43), B (n=159), and AB (n=99). The total discrepancy rate between serological and genetical typing reached 42.38% (128/302) (**Table 1**). ABO gene sequencing revealed that 302 candidates all carried weak ABO alleles, with 295 phenotypes belonging to weak ABO subgroups. A total of 41 genotypes were detected with the most frequent genotypes in genetically typed groups A, B, and AB were AelO1 (25.58%, 11/43), B(A)O1 (23.90%, 38/159), and A1Bw (22.22%, 22/99), respectively (**Figure 1**). In total, 49 kinds of weak ABO alleles were analyzed. The most frequent alleles were ABO*Aw.37 (gene frequency 22.22%, 18/81), ABO*Bw.03 (gene frequency 19.82%, 22/111), ABO*BA.02 (gene frequency 59.77%, 52/87), and ABO*cisAB.01 (gene frequency 77.27%, 17/22).

**Table 1:** The discrepancy between serotype and genotype.

Serotype	Individuals	Genotype	Phenotype	Individuals	Discrepancy rate (%)
O	19	AelO1, AelO2, AelO5	A	7	94.74
		BelO1, BelO2, BelO4	B	11	
		O1O7	O	1	
A	43	A1Bel, A2B*, AwB	AB	14	32.56
		A1A2, A2A2, A2O1, A2O2, A3O1, A3O2, AelO1, AelO2, AwO1, AwO2	A	29	
B	63	A1B3	AB	2	3.17
		BBw, B3O1, B3O2, BelO1, BelO2, BelO4, BwO1, BwO2, BwO6, BwO7	B	61	
AB	177	A2O1, AelO1	A	7	53.11
		B3O1, B(A)O1, B(A)O2, B(A)O56, B(A)B	B	87	
		A1Bw, A1B3, A2B, A3B, AelB, AwB, AwBw, B(A)A1, cisAB/O1, cisAB/O2, cisAB/A1, cisAB/B	AB	83	

*B stands for B.01, B.02, B.03 according to ISBT.

### The Detection of Irregular Antibody Intensity in 302 Transplant Candidates

Among the samples, 77.48% (n=234) containing irregular ABO antibodies were identified by ABO blood group typing and reversed typing. In the serologically typed A group, 86.05% (n=37) contained irregular anti-A antibodies; in the serologically typed B group, 68.25% (n=43) contained irregular anti-B antibodies; in the serologically typed AB group, 73.45% (n=130) contained irregular anti-A antibodies and 23.73% (n=42) contained irregular anti-B antibodies. Interestingly, in the serologically typed O group, we found one sample showing negative anti-A antibodies, which was confirmed as O1O7. The detailed antibody intensity is listed in **Table 2**.

**Table 2:** The serological characteristics of 302 candidates.

Serotype	Anti-A antibody %						Anti-B antibody %					
	Irregular antibody (n)	Intensity (n)					Irregular antibody (n)	Intensity (n)				
		±	+	++	+++	++++		±	+	++	+++	++++
O*(n=19)	94.74 (n=18)	26.32 (n=5)	10.53 (n=2)	47.37 (n=9)	5.26 (n=1)	5.26 (n=1)	100 (n=19)	52.63 (n=10)	5.26 (n=1)	42.11 (n=8)	0	0
A (n=43)	86.05 (n=37)	0	48.84 (n=21)	37.21 (n=16)	0	0	100	4.65 (n=2)	9.30 (n=4)	4.65 (n=2)	11.63 (n=5)	69.77 (n=30)
B (n=63)	100	0	0	0	28.57 (n=18)	71.43 (n=45)	68.25 (n=43)	52.38 (n=33)	15.87 (n=10)	0	0	0
AB* (n=177)	73.45 (n=130)	3.39 (n=6)	22.60 (n=40)	16.95 (n=30)	23.16 (n=41)	7.34 (n=13)	23.73 (n=42)	11.30 (n=20)	9.04 (n=16)	2.26 (n=4)	1.13 (n=2)	0

*One sample in blood group O does not contain anti-A antibody, and 5 samples in blood group AB contain both anti-A antibody and anti-B antibody.

### The Simulated Mismatching Rate in Donor Selection According to Serotype for 302 Transplant Candidates

**Figure 2:**
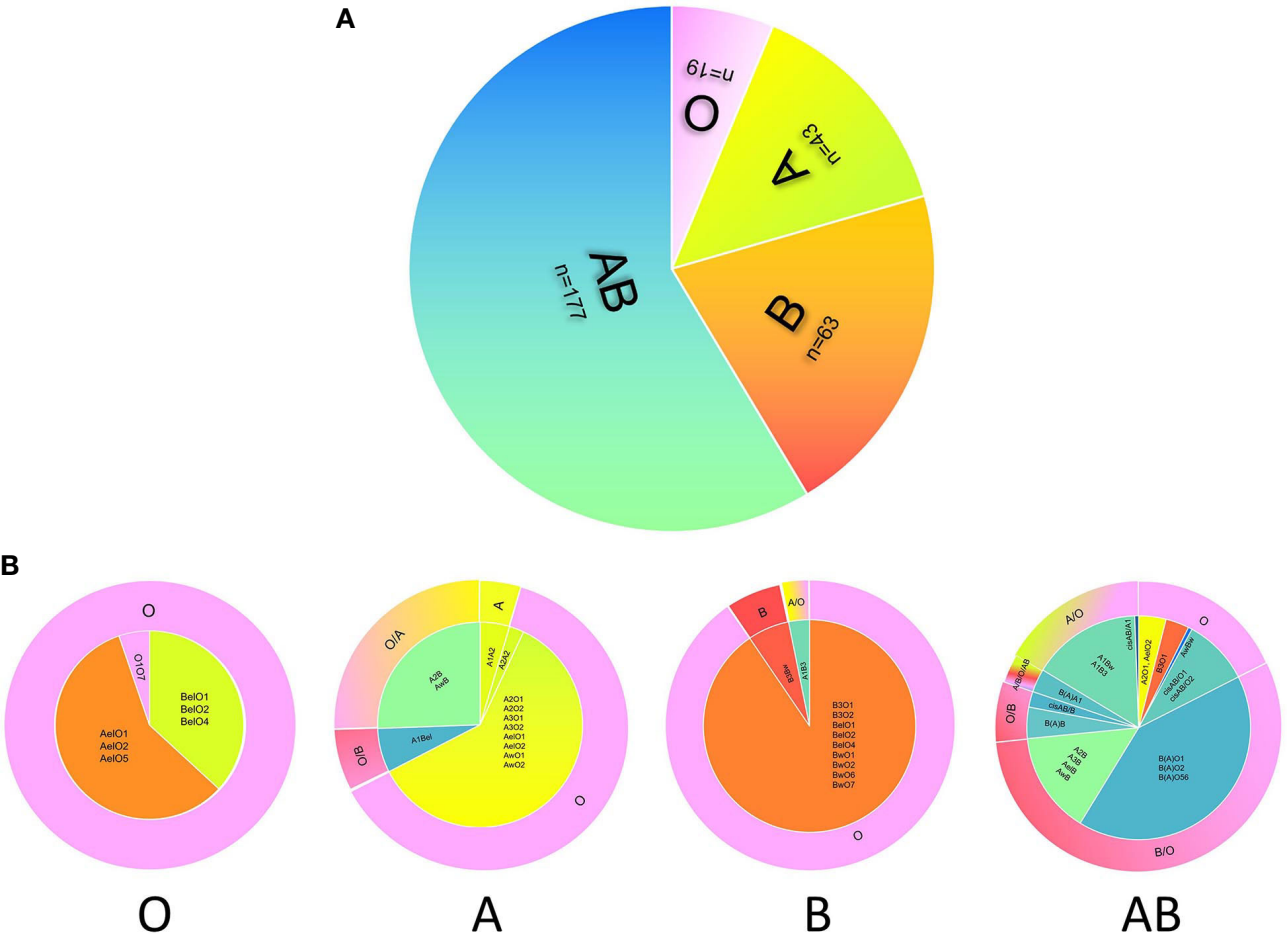
The discrepancy of donor selection according to serotype and genotype. The distribution of serotypes of 302 candidates with blood grouping discrepancies (**A**). Donor selection according to the serotype **(A)** or genotype **(B)** of candidate shows large difference. The inner circles of panel b show the distribution of genotypes in four blood groups, and the outer circles represent the best choice of donor blood type according to the genotype. For the outer circle, the part of candidate’s serotype consists with the donor blood type means ABO match, while the other part all means ABO mismatch. The color of pink, green, orange, and blue represent O, A, B, and AB respectively.

In this study, we analyzed donor selection according to serotype and genotype separately. Considering the ABO genotype and irregular blood antibodies, “ABO mismatch” was used to refer to a status in which a donor’s blood type was selected by a serotyping mismatch with the actual ABO genotype of a recipient with intensive blood antibodies. The mismatching rate of donor selection according to serotype was 88.74% (268/302), and the rates in serological O, A, B, and AB groups were 0% (0/19), 88.37% (38/43), 91.30% (59/63), and 96.61% (171/177), respectively (**Table 3** and **Figure 2**).

**Table 3:** The discrepancy of donor selection according to serotype and genotype.

Serotype	Genotype	Individuals	Donor selection		Match/mismatch	Mismatch%
			Serotype	Genotype		
O	AelO1, AelO2, AelO5	7	O	O	Match	
	BelO1, BelO2, BelO4	11	O	O	Match	
	O1O7	1	O	O	Match	
Sum		19				0 (0/19)
A	A1A2	2	A	A	Match	
	A2A2	1	A	O	Mismatch	
	A2O1, A2O2, A3O1, A3O2, AelO1, AelO2, AwO1, AwO2	26	A	O	Mismatch	
	A1Bel	3	A	O/A	Match	
	A2B*, AwB	11	A	O/B	Mismatch	
Sum		43				88.37 (38/43)
B	B3O1, B3O2, BelO1, BelO2, BelO4, BwO1, BwO2, BwO6, BwO7	57	B	O	Mismatch	
	BBw	4	B	B	Match	
	A1B3	2	B	A/O	Mismatch	
Sum		63				91.30 (59/63)
AB	A2O1, AelO1	7	AB	O	Mismatch	
	B3O1	6	AB	O	Mismatch	
	B(A)O1, B(A)O2, B(A)O56	73	AB	O/B	Mismatch	
	B(A)B	8	AB	B/O	Mismatch	
	AwBw	1	AB	O	Mismatch	
	A1Bw, A1B3	28	AB	O/A	Mismatch	
	A2B,A3B, AelB, AwB	26	AB	O/B	Mismatch	
	B(A)A1	6	AB	AB/O/A/B	Match	
	cisAB/O1, cisAB/O2	17	AB	O	Mismatch	
	cisAB/A1	1	AB	A/O	Mismatch	
	cisAB/B	4	AB	B/O	Mismatch	
Sum		177				96.61 (171/177)

*B stands for B.01, B.02, B.03 according to ISBT.

### Differences in Detection Rate of the Weak A Subgroup

We collected the weak A subgroup distribution identified by serological screening in healthy Chinese populations from previous researches (16–19), and the frequency was estimated to be 0.35%. Our data from 356 blood samples showed that 2.53% of blood type A kidney transplant candidates were identified as weak A subgroups by both serological and genetic test. Of these candidates, 1.97% (7/356) were A2 and 0.56% (2/356) were Aw, which were significantly higher than that of healthy Chinese people in previous studies (χ^2^ test, P<0.01). But it’s much lower than that of Caucasians (20%) (χ^2^ test, P<0.01) due to genetic differences among ethnic groups (20–22) (**Tables 4** and **5**).

**Table 4:** The distribution of weak A subgroup in different populations.

Population	Total A (n)	Weak A (n)	Weak A %	Lower	Upper	References
American	120	23	19.17%			C. Gassner et al.
American	84	16	19.05%			Nelson PW et al.
European			22.00%			Daniels G.
Chinese (Zhejiang)	8,707	36	0.41%	0.29%	0.57%	Ying Y et al.
Chinese (Nanjing)	7,839	24	0.31%	0.20%	0.46%	Chen Y et al.
Chinese (Shandong)	3,395	22	0.65%	0.41%	0.98%	Du Z et al.
Chinese (Guangdong)	6,664	16	0.24%	0.14%	0.39%	Xie J et al.
Chinese (sum)			0.35%	0.17%	0.54%	
Our study	356	9	2.53%			

**Table 5:** The distribution of A subtypes in 356 candidates.

Serotype	Phenotype	Genotype	Individuals	Relative frequency (%)	
A	A1	A1A1	51	14.33	97.47
		A1A2	2	0.56	
		A1O1	181	50.84	
		A1O2	108	30.34	
		A1O4	5	1.40	
	Non A1	A2O1	3	0.84	2.53
		A2O2	4	1.12	
		AwO2	2	0.56	

## Discussion

We revealed the high risk of blood type misjudgment and genetically ABO-mismatched transplantation based on both serological and genetic test. Improved precision of ABO genotyping is crucial for successful kidney transplantation and reasonable organ allocation.

Studies have reported the success of kidney transplantation across the blood group barrier (23, 24), but the risks for severe infection, antibody-mediated rejection, and postoperative bleeding were all increased in an ABO-incompatible patient groups. Britta Eiz-Vesper *et al.* showed that different ABO genotypes hiding behind identical phenotypes encode for different sets of glycosyltransferases, which provide a source for minor histocompatibility antigens in allogeneic peripheral blood progenitor cell transplantation. Thus, considering allelic ABO sequences, at least 15% of all phenotypically ABO-matched transplant pairs can be expected to have genotype constellations relevant to graft-*versus*-host disease (GVHD) (25). Ushigome *et al. *revealed a higher risk of transplant glomerulopathy caused by chronic or active antibody-mediated rejection within 1 year after ABOi kidney transplantation (26). Dashkova *et al*. examined the irregular anti-A1 antibody-containing serum from 43 samples with A2 and A2B blood groups, which might be the reason for posttransfusion reactions or complications in recipients (27). These findings indicated that ABO incompatibility and anti-A1/A/B titers may be the strongest risk factors for graft rejection after kidney transplantation.

In the present study, ABO gene sequencing revealed that all the 302 candidates carried weak ABO alleles, leading to grouping discrepancies observed in the reciprocal serotyping. Therefore, the only serological test bears a serious risk for erroneous typing of ABO group, especially for blood groups O and AB based on the results of our cohort. Accordingly, our findings indicated that misjudgment also existed in donor blood typing, such as mistaking A or B subgroup for O, resulting in ABO-mismatched transplantation. Noticeably, 77.48% of the candidates carried irregular ABO antibodies, which can occur naturally or as a result of a previous blood transfusion, pregnancy, and chronic diseases (28). These relevant clinical events were associated with aberrant ABO gene expression and the production of blood group antibodies (29). Considering the complete expression of the ABO blood group antigen in the kidney (30–32), the irritation of the irregular antibody pre-transplantation would complicate blood group typing and, ultimately, donor selection.

In our study we evaluated ABO genotype and blood antibody intensity and found that the mismatch rate of donor selection by serotype was high for all blood types (88.37-96.61%) except type O. Clinically, type AB candidates have the easiest outlook to choose a donor with a relatively short waiting time (23). However, our data demonstrated that AB candidates had the highest probability (96.61%) of ABO-mismatched kidney transplantation. More importantly, 17.51% (31/177) of AB candidates analyzed in our study were strongly recommended to choose a type O graft for better long-term outcome. Therefore, the negligence of ABO genotyping will increase the risk of delayed graft function and rejection. ABOi living donor kidney transplantation was pioneered in Japan with excellent reported outcomes (33). The immunosuppressive regimen was adjusted and a splenectomy was completed according to serum ABO antibodies. The risk of early rejection and severe infection was higher in ABOi groups (34). Besides, it is a common phenomenon that blood transfusion for patients with renal anemia is lack of standardization in primary medical institution, which further increased the possibility of errors in serotyping later. We presented simulated mismatch rates here to estimate the huge risk in donor selection by serotyping only.

The A2 allele was characterized by a single base deletion (1061del C) compared with the A102 allele (35, 36), resulting in both a qualitative and quantitative difference between A1 and A2. The transferase activity of A1 is 5- to 10-fold greater than that of A2 and is much higher than that of other weak A subgroups (37). Therefore, A1-to-A2 transplantation can induce antibody-mediated rejection, whereas A2 and other weak A subgroups can be an alternative choice for type O candidates due to decreased A antigen expression on the renal endothelial cells (38, 39). In our previous report, a recipient experienced four AR episodes within 3 to 10 months post-transplantation, and graft function continued to decrease progressively after empirical administration of high dose methylprednisolone. The patient experienced graft loss 1 year after transplantation. Posttransplant ABO genotyping showed an A1O1-to-A2O1 mismatch, which was further confirmed by the antibody detection of anti-A1 in the serum with negative panel reactive antibody (38, 39).

Our results also showed that the actual weak A subgroup was larger than previously estimated by serotype screening. A donor graft in the weak ABO subgroup may not be precisely allocated if without genotyping. The United Network for Organ Sharing (UNOS) advocates A2-to-O/B transplantation to shorten the waiting time for type O/B candidates (12). However, due to the rareness of A2 individuals in our country, it cannot be the common alternative for the O blood group. Therefore, we recommend precise ABO genotyping prior to kidney transplantation to guarantee genotypically ABO-matched transplants as well as rational use of grafts in weak ABO subgroups.

## Conclusions

The state of shrinking supply of corpus organs maintains. Due to the frequent occurrence of irregular ABO antibodies and the discrepancy between serotyping and genotyping, precise ABO genotyping protocols are urgently required for donor selection in kidney transplant candidates in order to reduce the risk of acute rejection and improve the rationality of organ allocation.

## Data Availability Statement

The original contributions presented in the study are included in the article/**Supplementary Material**. Further inquiries can be directed to the corresponding authors.

## Ethics Statement

The studies involving human participants were reviewed and approved by Ethics committees of Nanfang Hospital, Southern Medical University. The patients/participants provided their written informed consent to participate in this study (NFEC-2019-172).

## Author Contributions

YZ, YCW, LX, and YM designed the study. DL, NC, YHW, KQ, and SW carried out experiments. WD, HL, and SF analyzed the data. YCW made figures. YZ, JX, HN, and FX drafted and revised the paper. All authors contributed to the article and approved the submitted version.

## Funding

This study was funded by the Science and Technology Planning Project of Guangzhou (Grant No. 201803010109), Natural Science Foundation of Guangdong Province (Grant No. 2020A1515010674), the President Funding of Nanfang Hospital (Grant No. 2018B009, 2018C003) and College Students’ Innovative Entrepreneurial Training Plan Program (Grant No. 201812121148, X202012121239, 202012121046).

## Conflict of Interest

The authors declare that the research was conducted in the absence of any commercial or financial relationships that could be constructed as a potential conflict of interest.
