# Supplementary material for: The Potential Significance of ABO Genotyping for Donor Selection in Kidney Transplantation
Source: Front Immunol. 2020 Nov 19;11:608716. doi: 10.3389/fimmu.2020.608716 (PMC7710857; doi:10.3389/fimmu.2020.608716)
Supplement: Supplementary file 1 [file Table_1.docx]

Supplementary Table Ⅰ. Sequence-specific primers

| Gene |  | Primer sequence (5′ to 3′) | Size (bp) |
| --- | --- | --- | --- |
| ABO-EXON1 | sense | CTCTGTCCCCTCCCGTGTTC | 273 |
|  | anti-sense | CTGAAGCCTCGCAGCCCT |  |
| ABO-EXON2 | sense | GCAGGTGAGAGAAGGAGGGT | 266 |
|  | anti-sense | AGCTGGACGCAGGCAATAAC |  |
| ABO-EXON3 | sense | CACCGGGAACTCGTGTGCTC | 204 |
|  | anti-sense | ATGGATGCTCCACCTGCTCT |  |
| ABO-EXON4 | sense | GTTTCTGGTGGCCTCTGCTC | 281 |
|  | anti-sense | GCTCCCACATGCTTCTGTCC |  |
| ABO-EXON5 | sense | CTGCATCCCACGCTTTCCAT | 318 |
|  | anti-sense | GCAGGGGCTTTGGAGAACAA |  |
| ABO-EXON6 | sense | GGAATGATTTGCCCGGTTGG | 390 |
|  | anti-sense | TCAATGTCCACAGTCACTCGC |  |
| ABO-EXON7 | sense | CCCCGTCCGCCTGCCTTGCA | 837 |
|  | anti-sense | GGGCCTAGGCTTCAGTTACTC |  |

Supplementary Table Ⅱ. The distribution of weak ABO alleles in 302 candidates

| N. | Allele | Individuals | Relative frequency(%) | Genotype |
| --- | --- | --- | --- | --- |
| 1 | ABO*Aw.37 | 18 | 5.96 | AwO1,AwO2,AwB§,AwBw^‡^ |
|  | ABO*A2.01 | 13 | 4.30 | A2O1, A2O2, A2B§ |
|  | ABO*A2.05 | 12 | 3.97 | A1A2, A2A2, A2O1, A2O2, A2B§ |
|  | ABO*Ael.05 | 9 | 2.98 | AelO1, AelO2 |
|  | ABO*A3.07 | 6 | 1.99 | A3O1,A3O2, A3B§ |
|  | ABO*Ael.02 | 4 | 1.32 | AelO1, AelO2 |
|  | ABO*A2.13 | 3 | 0.99 | A2O1 |
|  | ABO*Aw.35 | 3 | 0.99 | AwO1, AwO2 |
|  | ABO*Aw.43 | 3 | 0.99 | AwO1,AwB§ |
|  | ABO*A2.09 | 2 | 0.66 | A2B§ |
|  | ABO*A2.21^†^ | 1 | 0.33 | A2O1 |
|  | ABO*A2.23^†^ | 1 | 0.33 | A2B§ |
|  | ABO*Ael.01 | 1 | 0.33 | AelO1 |
|  | ABO*Ael.10^†^ | 1 | 0.33 | AelO1 |
|  | ABO*Ael.13^†^ | 1 | 0.33 | AelO5^‡^ |
|  | ABO*Aw.30 | 1 | 0.33 | AwO1 |
|  | ABO*Aw.31.02-05 | 1 | 0.33 | AwO2 |
|  | ABO*Ax.22^†^ | 1 | 0.33 | AxB§ |
| 2 | ABO*Bw.03 | 22 | 7.28 | A1Bw,BwO1,BwO2 |
|  | ABO*Bw.12 | 19 | 6.29 | A1Bw,BwO1,BwO2,BwO6^$^ |
|  | ABO*Bel.06 | 16 | 5.30 | BelO1,BelO2,BelO4^‡^ |
|  | ABO*B3.03 | 9 | 2.98 | A1B3,B3O1,B3O2 |
|  | ABO*Bel.03 | 9 | 2.98 | A1Bel,BelO1,BelO2 |
|  | ABO*Bw.37^†^ | 7 | 2.32 | A1Bw,BBw§ |
|  | ABO*B3.05 | 5 | 1.66 | A1B3,B3O1 |
|  | ABO*Bw.07 | 4 | 1.32 | A1Bw |
|  | ABO*Bw.11 | 3 | 0.99 | A1Bw,BwO1 |
|  | ABO*Bel.09^†^ | 2 | 0.66 | BelO2 |
|  | ABO*Bel.11^†^ | 2 | 0.66 | BelO1,BelO4^‡^ |
|  | ABO*Bw.14 | 2 | 0.66 | BwO1,BwO7 |
|  | ABO*B3.09^†^ | 1 | 0.33 | A1B3 |
|  | ABO*Bw.01 | 1 | 0.33 | BwO1 |
|  | ABO*Bw.16 | 1 | 0.33 | A1Bw |
|  | ABO*Bw.17 | 1 | 0.33 | BwO1 |
|  | ABO*Bw.22 | 1 | 0.33 | BwO1 |
|  | ABO*Bw.31 | 1 | 0.33 | BwO1 |
|  | ABO*Bw.32 | 1 | 0.33 | A1Bw |
|  | ABO*Bw.35^†^ | 1 | 0.33 | A1Bw |
|  | ABO*Bx.12^†^ | 1 | 0.33 | A1Bx |
|  | ABO*B1.10^†^ | 1 | 0.33 | A1Bw |
|  | ABO*B1.19^†^ | 1 | 0.33 | BwO2 |
| 3 | ABO*BA.02 | 52 | 17.22 | B(A)A1,B(A)B§,B(A)O1,B(A)O2,B(A)O56^‡^ |
|  | ABO*BA.04 | 34 | 11.26 | B(A)A1,B(A)B§,B(A)O1,B(A)O2 |
|  | ABO*BA.03 | 1 | 0.33 | B(A)O1 |
| 4 | ABO*cisAB.01 | 17 | 5.63 | cisAB/A1,cisAB/B§,cisAB/O1,cisAB/O2 |
|  | ABO*cisAB.05 | 3 | 0.99 | cisAB/O1,cisAB/O2 |
|  | ABO*cisAB.06 | 1 | 0.33 | cisAB/O1 |
|  | ABO*cisAB.09 | 1 | 0.33 | cisAB/B§ |
| 5 | ABO*O.07 | 1 | 0.33 | O1O7 |
| †Thirteen alleles haven't been named by ISBT, and they are named according to NCBI. ‡Six samples contain two weak ABO subgroup alleles as Ael.13/O05, Aw.37/Bw.14, Bel.06/O.04, Bel.11/O.04, Bw.12/O.06, BA.02/O.56. §B stands for B.01, B.02, B.03 according to ISBT. | | | | |
